# Influence of Patella thickness on Patellofemoral pressure in total knee Arthroplasty

**DOI:** 10.1186/s12891-021-04175-y

**Published:** 2021-03-23

**Authors:** Hidenori Tanikawa, Mitsunori Tada, Ryo Ogawa, Kengo Harato, Yasuo Niki, Shu Kobayashi, Takeo Nagura

**Affiliations:** 1 Department of Orthopedic Surgery, Shiroi Seijinkai Hospital, 3-25-2 Sasazuka, Shiroi-shi, Chiba, 270-1426 Japan; 2 Digital Human Research Team, Artificial Intelligence Research Center, National Institute of Advanced Industrial Science and Technology, Tokyo, Japan; 3 Department of Orthopedic Surgery, Keio University School of Medicine, Tokyo, Japan; 4 Department of Clinical Biomechanics, Keio University, Tokyo, Japan

**Keywords:** Total knee arthroplasty, Patellofemoral joint, Biomechanics

## Abstract

**Background:**

Patellofemoral complications are one of the major issues after total knee arthroplasty (TKA). Excessive patellofemoral joint pressure is associated with complications after TKA surgery, and the amount of patellar osteotomy has a direct effect on patellofemoral joint pressure. The purpose of this study was to evaluate the influence of patella thickness on patellofemoral pressure in TKA.

**Methods:**

Five freshly frozen cadavers were operated with a custom-made Stryker posterior stabilizing type knee joint prosthesis. Patellofemoral joint pressure was measured using a pressure sensor, with the knee joint flexed from 90 to 110 degrees, and with patellar thickness of − 2 mm to + 4 mm.

**Results:**

Increasing or decreasing patellar thickness significantly increased or decreased patellofemoral pressure. Regarding knee flexion angle, patellofemoral pressure increased with increasing patellar thickness at all flexion angles, but the pressure increase was greatest at 90 degrees of knee flexion and smallest at 110 degrees.

**Conclusions:**

The amount of patellar osteotomy influences the patellofemoral pressure. Surgeons should avoid increasing patella thickness, since the resulting increased patellofemoral pressure may reduce knee joint function.

## Background

Knee arthroplasty (TKA) is a very useful treatment for knee osteoarthritis and rheumatoid arthritis. Patellofemoral problems are one of the common postoperative complications and may result in revision surgery [1]. It has also been reported that 4–12% of all TKA revisions are due to the patellofemoral joint, such as anterior knee pain, loosening of the patellar component, and patellar fracture [2]. Five to 45% of post-TKA patients complain of residual anterior knee pain [3, 4]. Although details about the cause of anterior knee pain are still unknown, low patellofemoral pressure are preferable because high pressure may cause anterior knee pain [5, 6].

Tibiofemoral and patellofemoral joint pressures were difficult to measure in the past. The development of miniature sensors called MEMS (Micro Electro Mechanical Systems) has enabled researchers to measure intraarticular pressures in these joints. In 2012, a TKA insert trial with a pressure sensor built-in was used in the first publication on obtaining soft tissue balance using intraoperative tibiofemoral joint pressure as an indicator [7].

With respect to patellofemoral joint pressure, it is still in its infancy and no commercially available device capable of measuring patellofemoral joint pressure is available on the market to date. Various studies have been conducted on patellofemoral joint pressure by incorporating thin pressure sensors into implants and using finite element methods and inverse dynamics to analyze the pressure at the patellofemoral joint [8–16]. The clinical significance of patellofemoral joint pressure remains unclear, but it may be associated with, for example, implant wear, loosening, and anterior knee joint pain [5, 6, 14]. In the present study, we focused on patellofemoral pressure and investigated the influence of patellar cut volume on patellofemoral pressure.

## Methods

Five fresh frozen human cadaver specimens (3 males and 2 females) were examined. None of the specimens had any skeletal or articular pathology in lower extremities. The cadavers of this study were provided by the clinical anatomy laboratory of Keio University. Prior to the experiment, all study protocols were approved by the ethics review board at our institution, and the experiment was conducted according to the Guidelines for Cadaver Dissection in Education and Research of Clinical Medicine. The consent to use the cadavers was obtained from the patient before death.

**Fig. 1:**
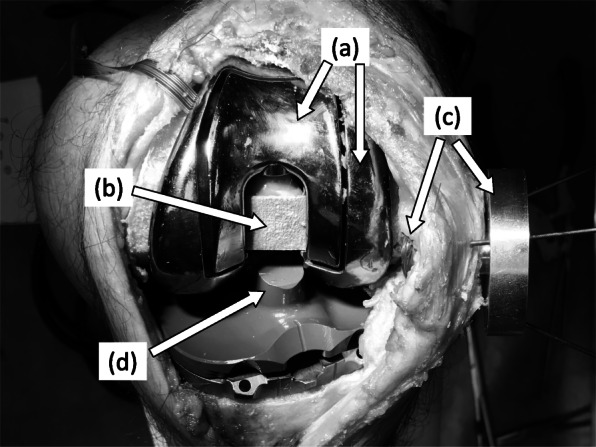
**a** Custom made femoral component with (**b**) a built-in pressure sensor, **c** adjustable thickness patellar component, and (**d**) a trial insert with a 2-mm shorter post height than normal

**Fig. 2:**
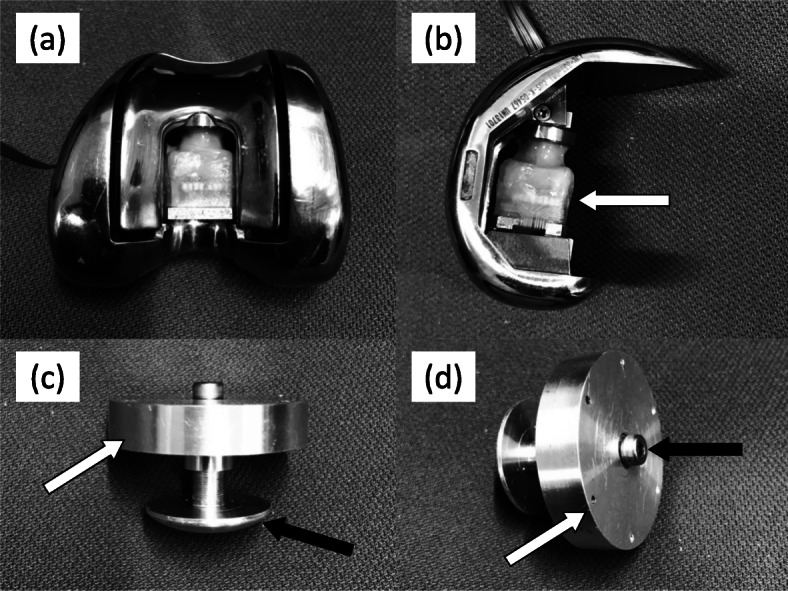
**a** Frontal view and (**b**) lateral view of a custom-made femoral component with a built-in pressure sensor (white arrow). **c** An adjustable thickness patellar component was composed of the base part (white arrow) and the patella part (black arrow). **d** The base part (white arrow) was fixed on a patella with k-wires, then a patella thickness was adjusted by rotating a screw hole of the patella part (black arrow)

To measure patellofemoral pressure in various patella thickness, we used a femoral component that incorporates a 6-axis pressure sensor (Leptrino Co., Nagano, Japan) and a patella component that can be adjusted to various thicknesses (Fig. 1). The 6-axis pressure sensor was 20 mm*20 mm*30 mm in size. We isolated the part of the femoral component where it meets the patella and placed the 6-axis pressure sensor inside the femoral component, therefore the patellofemoral pressure measured in this study was a total of compressive force occurs on the isolated part of the femoral component (Fig. 2). The sensor was placed in the space of the box cut. Because of the loss of space in the box cut, it was impossible to extend the knee joint with this femoral component. The post of a normal insert came into contact with the pressure sensor, so we made an insert with a 2-mm shorter post height. Using this insert and a pressure-sensor-containing femoral component, patellofemoral joint pressure at a 90-degree knee flexion angle and above can be measured. The adjustable thickness patellar component consists of an articular surface and a base. The diameter of the patella component was 29 mm and the original thickness was 8 mm. After the patella was osteotomized, a hole was drilled in the center of the patella and the base was fixed to the ventral side of the patella. The articular surface was attached to the base and it can be moved up and down by turning a screw. Since the screw hole is located on the ventral side of the patella, the thickness of the patella can be adjusted with the articular capsule sutured (Figs. 1, 2).

A standard TKA surgery was performed using the Triathlon TKA system (Stryker, Kalamazoo, MI, USA). To keep the joint line, we cut the distal femur 8 mm from the bone surface and cut the proximal tibia 9 mm from the lateral surface of tibia. The surgery was performed according to the company surgical guidelines using the customized patellar component, femoral component, and posterior stabilized type insert. We measured the patellofemoral joint pressure with the patella osteotomy volume 2 mm more than the patellar component thickness. Since the normal patellar component thickness was 8 mm, the patella osteotomy volume was 10 mm in this study. We first measured patellofemoral joint pressure at 90 degrees of knee flexion and 8 mm of patellar thickness. Then, while keeping the angle of knee joint flexion constant, we measured the pressure at patella thickness of -2 mm, + 2 mm, and + 4 mm. In addition, measurements were taken with the knee joint flexion angle of 100 degrees and 110 degrees. We measured the patellofemoral pressure in an unloaded position in this study.

Statistical comparison of the results was performed using SPSS version 17.0 software (SPSS Inc., Chicago, IL). Means and standard deviations were used to describe the data. Statistical differences between different conditions were analyzed using one-way analysis of variance on ranks with post hoc Dunnett’s test (multiple comparisons versus a control).

## Results

**Fig. 3:**
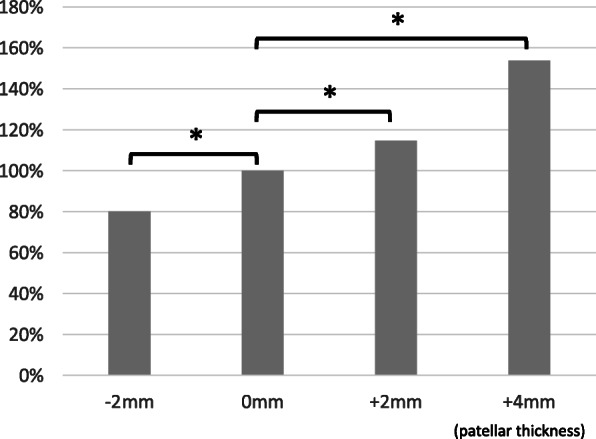
Patellofemoral joint pressure when the patellar thickness was changed. Values are normalized by the data with standard patella osteotomy (0 mm). * indicates significant difference (*P* < 0.05)

**Fig. 4:**
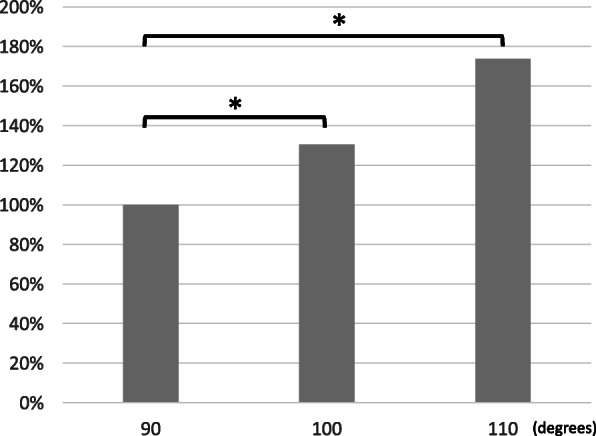
Patellofemoral joint pressure when the knee joint angle was changed. Values are normalized by the data of 90 degrees knee flexion. * indicates significant difference (*P* < 0.05)

Increasing or decreasing patellar thickness significantly increased or decreased patellofemoral pressure (− 2 mm: 80.0%, 0 mm: 100%, + 2 mm: 114.8%, + 4 mm: 153.8%, Fig. 3). Regarding knee flexion angle, patellofemoral pressure increased with increasing patellar thickness at all flexion angles, but the pressure increase was greatest at 90 degrees of knee flexion and smallest at 110 degrees. Compared to the patellofemoral pressure at 90 degrees of knee flexion, the patellofemoral pressure increased by 30.5% at 100 degrees and 73.8% at 110 degrees (Fig. 4).

## Discussion

The results of this study showed that a 2 mm increase or decrease in patellar thickness resulted in an approximately 20% increase or decrease in the patellofemoral pressure. The advantage of this study is very accurate measurements of patellofemoral pressure with various patella component thicknesses. There are various methods for measuring patellofemoral joint pressure [8–16]. In the method of measuring pressure by placing a sensor between the patellofemoral joint, the pressure sensor may shift, and the patellofemoral joint pressure may be increased due to the thickness of the pressure sensor [8, 9, 11, 12]. On the other hand, with a pressure sensor built into the patella, patellofemoral joint pressure can be measured accurately [13–15]. However, because the osteotomy and joint capsule sutures are redone when changing the patellar thickness, the conditions are not constant. In our method, the sensor is built into the femoral component, and the patellofemoral joint pressure can be measured without inserting a thick sensor in the patellofemoral joint. Furthermore, since the thickness of the patellar component is adjustable, patellofemoral joint pressures at various patellar thicknesses can be made under identical conditions without re-suturing of the joint capsule. Thus, this study has the advantage of obtaining very accurate measurements.

Although previous reports have reported that a 2 mm increase in patellar thickness resulted in a 2.7-fold increase in patellofemoral pressure [17], the increase in patellofemoral pressure in the present experiment was 14.8%. We believe that this difference can be attributed to differences in measurement methods. The amount of patellar osteotomy is associated with postoperative knee joint function, and it has been reported that for every 1 mm increase in patellar thickness, joint range of motion is reduced by 3 degrees [18]. A study researching influence of patellofemoral pressure on patient-reported outcome has reported that the patellofemoral pressure at 140° of flexion was negatively correlated with patient satisfaction and Forgotten Joint Score-12, and that the patellofemoral pressure at 60° of flexion was negatively correlated with the patella score [14].

In general, the osteotomy volume of the patella should be the same as the thickness of the patellar component. The results of the present study showed that resection of an additional 2 mm of patella reduced patellofemoral joint pressure by 20%. However, increasing the osteotomy volume to decrease patellofemoral joint pressure is not recommended and should be done cautiously.

Thinning the patella by more than 2 mm compared to the original patella thickness has been reported to increase the risk of postoperative patellar clunk/crepitus [19]. Also, if the native patella thickness is thin, there is a risk of patellar fracture when the patella thickness after osteotomy is less than 12 mm [20]. In such cases, the choice of non-patellar replacement or the use of an in-lay type or a thinner patella component should be considered. Patellofemoral load pressure does not only increase with patellar thickness, but also with an anterior position of the femoral implant. Thus, it is necessary to resect the femur near flush to the anterior cortex to avoid patellofemoral overstuffing and resulting unsatisfactory flexion.

Our study has several limitations. First, the sample size was small so a type II error might not be fully excluded in our study. However, the number of cadavers used in this study, five, seems to be a reasonable number compared to similar studies in the past [5, 8, 9, 12]. Second, we were not able to simulate physiological load of the quadriceps or hamstring muscles in this study. It is inferred that the actual patellofemoral pressure is much higher than in our experiment due to the action of the muscles and the weight bearing. Highly accurate and ethically acceptable measurement of the knee under weight-bearing conditions in vivo would be ideal. Finally, we have only experimented with one type of knee joint prosthesis in the present study. The knee joint prosthesis used in this study has a single-radius shape for the femoral implant and a disc shape for the patella. Patellofemoral joint pressure may be different with an implant with a multi-radius shape of the femoral implant or an anatomical shape of the patella.

## Conclusions

The results of this study showed that a 2 mm increase or decrease in patellar thickness resulted in an approximately 20% increase or decrease in the patellofemoral pressure. Care should be taken not to increase the thickness during patellar replacement since increased patellofemoral pressure may reduce knee joint function.

## Data Availability

The datasets used and/or analyzed during the current study are available from the corresponding author on reasonable request.

## Abbreviation

TKA: Total knee arthroplasty
